# 115. Post-licensure Safety Study of New-onset Immune-mediated Diseases, Herpes Zoster, and Anaphylaxis in Adult Recipients of HepB-CPG Vaccine Versus HepB-alum Vaccine

**DOI:** 10.1093/ofid/ofac492.193

**Published:** 2022-12-15

**Authors:** Bradley Ackerson, Lina S Sy, Jeff Slezak, Lei Qian, Kristi Reynolds, Runxin Huang, Zendi Solano, William Towner, Sijia Qiu, Sarah Simmons, Steven Jacobsen, Katia J Bruxvoort

**Affiliations:** Kaiser Permanente Southern California, Pasadena, California; Kaiser Permanente Southern California, Pasadena, California; Kaiser Permanente Southern California, Pasadena, California; Kaiser Permanente Southern California, Pasadena, California; Kaiser Permanente Southern California, Pasadena, California; Kaiser Permanente Southern California, Pasadena, California; Kaiser Permanente Southern California, Pasadena, California; Kaiser Permanente Southern California, Pasadena, California; Kaiser Permanente Southern California, Pasadena, California; Kaiser Permanente Southern California, Pasadena, California; Marshfield Clinic Research Institute, Iowa City, Iowa; University of Alabama at Birmingham, Birmingham, Alabama

## Abstract

**Background:**

HepB-CpG (Heplisav-B; Dynavax) is a licensed hepatitis B vaccine with a novel adjuvant that requires only 2 doses (0, 1 month) compared to a 3-dose (0, 1, 6 months) HepB-alum vaccine (Engerix-B; GlaxoSmithKline). Monitoring of safety outcomes following receipt of vaccines with novel adjuvants is important. Hence, as part of an FDA postmarketing commitment, we compared the incidence of new-onset immune-mediated diseases, herpes zoster (HZ), and anaphylaxis among recipients of HepB-CpG versus HepB-alum at Kaiser Permanente Southern California (KPSC).

**Methods:**

This cohort study included adults not on dialysis who received ≥1 dose of a hepatitis B vaccine from 8/7/2018 to 10/31/2019, during which HepB-CpG was routinely administered in 7 of 15 KPSC medical centers while HepB-alum was administered at the other 8 medical centers. Recipients of HepB-CpG or HepB-alum were followed through electronic health records for 13 months after receipt of the first dose during the vaccine accrual period for occurrence of pre-specified new-onset immune-mediated diseases, HZ, and anaphylaxis identified using diagnosis codes. Incidence rates were compared using Poisson regression with inverse probability of treatment weighting when there was 80% power to detect a relative risk (RR) of 5 for anaphylaxis and a RR of 3 for all other outcomes

**Results:**

There were 31,183 HepB-CpG and 38,442 HepB-alum recipients (overall 49.0% female, 48.5% ≥50 years of age, and 49.6% Hispanic). Among immune-mediated events that occurred frequently enough for formal comparison, rates among HepB-CpG versus Hep-B-alum recipients were similar except for rheumatoid arthritis (RA) (adjusted RR 1.53 [95% CI: 1.07, 2.18]). (Table 1) After adjudication of new-onset RA, the adjusted RR was 0.93 (0.34, 2.49). (Table 2) The adjusted RR for HZ was 1.06 (0.89, 1.27). Anaphylaxis occurred in 0 HepB-CpG and 2 HepB-alum recipients.

**Figure:**
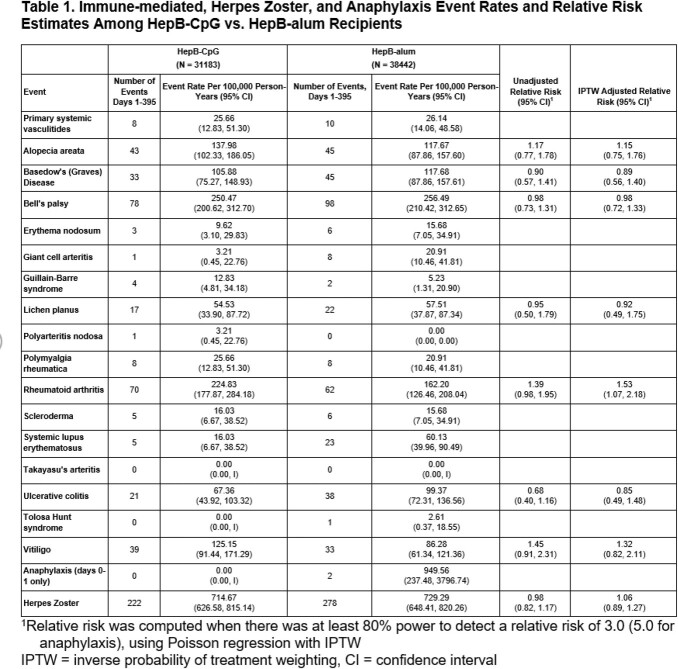

**Figure:**
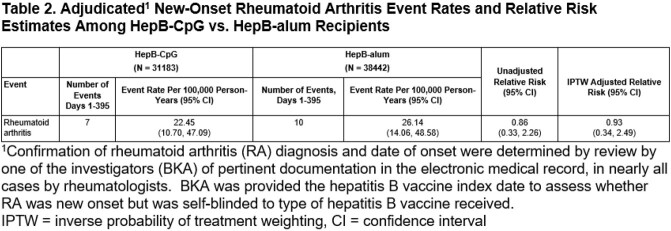

**Conclusion:**

These data suggest no safety concerns for HepB-CpG compared to HepB-alum for select immune-mediated diseases, HZ, or anaphylaxis in this observational study of over 69,000 recipients of hepatitis B vaccines.

**Disclosures:**

**Bradley Ackerson, MD**, Dynavax: Grant/Research Support|Glaxosmithkline: Grant/Research Support|Moderna: Grant/Research Support|Pfizer: Grant/Research Support|Seqirus: Grant/Research Support **Lina S. Sy, MPH**, Dynavax: Grant/Research Support|Glaxosmithkline: Grant/Research Support|Moderna: Grant/Research Support|Seqirus: Grant/Research Support **Jeff Slezak, MS**, ALK, Inc.: Grant/Research Support|Dynavax: Grant/Research Support|Novavax, Inc.: Grant/Research Support|Pfizer, Inc.: Grant/Research Support **Lei Qian, PhD**, Dynavax: Grant/Research Support|Glaxosmithkline: Grant/Research Support|Moderna: Grant/Research Support **Kristi Reynolds, PhD**, Amgen: Grant/Research Support|Dynavax: Grant/Research Support|Merck: Grant/Research Support|Novartis: Grant/Research Support **Runxin Huang, MS**, Dynavax: Grant/Research Support **Zendi Solano, BS**, Dynavax: Grant/Research Support|Gilead: Grant/Research Support|GlaxoSmithKline: Grant/Research Support **William Towner, MD**, Dynavax: Grant/Research Support|Gilead: Grant/Research Support|Merck: Grant/Research Support|Moderna: Grant/Research Support|Pfizer: Grant/Research Support|ViiV: Grant/Research Support **Sijia Qiu, MS**, Dynavax: Grant/Research Support|Moderna: Grant/Research Support **Sarah Simmons, MPH**, Dynavax: Grant/Research Support|Glaxo-Smith Kline: Grant/Research Support|Pfizer: Grant/Research Support **Steven Jacobsen, MD**, Dynavax: Grant/Research Support **Katia J. Bruxvoort, PhD, MPH**, Dynavax: Grant/Research Support|Gilead: Grant/Research Support|Glaxosmithkline: Grant/Research Support|Moderna: Grant/Research Support|Pfizer: Grant/Research Support|Seqirus: Grant/Research Support.

